# Alginate-encapsulated enzymes mitigate deoxynivalenol-induced hepatic mitochondrial dysfunction and glycolithocholic acid accumulation in piglets

**DOI:** 10.1186/s40104-026-01484-x

**Published:** 2026-09-05

**Authors:** Wei He, Xiangyu Huo, Xiaolu Wen, Shuting Cao, Zongyong Jiang, Baoming Shi, Li Wang

**Affiliations:** 1 State Key Laboratory of Swine and Poultry Breeding, Key Laboratory of Animal Nutrition and Feed Science in South China, Ministry of Agriculture and Rural Affairs; Guangdong Provincial Key Laboratory of Animal Breeding and Nutrition, Institute of Animal Science, Guangdong Academy of Agricultural Sciences, Guangzhou, 510640 China; 2 College of Animal Science and Technology, Northeast Agricultural University, Harbin, 150030 China

**Keywords:** Alginate, Deoxynivalenol, Enzyme, Glycolithocholic acid, Liver, Mitochondria

## Abstract

**Background:**

Deoxynivalenol (DON), a mycotoxin commonly found in grains and feed, poses a serious threat to animal and public health. This study aimed to investigate the underlying mechanisms of DON-induced liver damage in piglets and to elucidate the potential of alginate-encapsulated enzyme in mitigating hepatotoxicity in vivo.

**Results:**

The results indicated that DON exposure induced growth retardation and hepatic mitochondrial damage in piglets, which were effectively rescued by DON-degrading enzymes (DDE) supplementation. Hepatic transcriptomic profiling revealed that DON induced downregulation of oxidative phosphorylation‑related genes and ATP-binding cassette subfamily B member 4 (ABCB4), accompanied by the upregulation of the chemokine CCL21 and the adhesion molecule VCAM1. In contrast, no downregulation of oxidative phosphorylation‑related genes or aberrant expression of the aforementioned factors was observed in the DDE treatment group. Meanwhile, DON promotes T cell recruitment and their differentiation into the pro-inflammatory Th17 subset by inducing NF-κB-mediated expression of IL-1β and CCL21, thereby amplifying the inflammatory response. Further analysis revealed that DON exposure induced metabolic dysfunction of lysophospholipids and glycolithocholic acid (GLCA) in the liver and suppressed farnesoid X receptor (FXR) expression. Mechanistically, the downregulation of ABCB4 by DON was associated with reduced expression of PPARγ coactivator-1α (PGC-1α) and FXR, suggesting a potential link involving the PGC-1α/FXR axis. This downregulation results in the accumulation of GLCA, thereby exacerbating hepatic damage. Notably, alginate-encapsulated DDE effectively reversed the DON-induced GLCA metabolic dysfunction and the Th17 immune response.

**Conclusions:**

Alginate-encapsulated DDE effectively mitigated DON-induced hepatic mitochondrial damage, T cell enrichment, and impaired bile acid metabolism in piglets. This study suggests a potential ABCB4‑dependent mechanism underlying DON-induced hepatotoxicity and provides a promising enzymatic strategy for mitigating DON contamination in vivo. However, further studies are required to clarify the potential mechanism of DON-induced hepatotoxicity.

**Graphical Abstract:**

**Figure:**
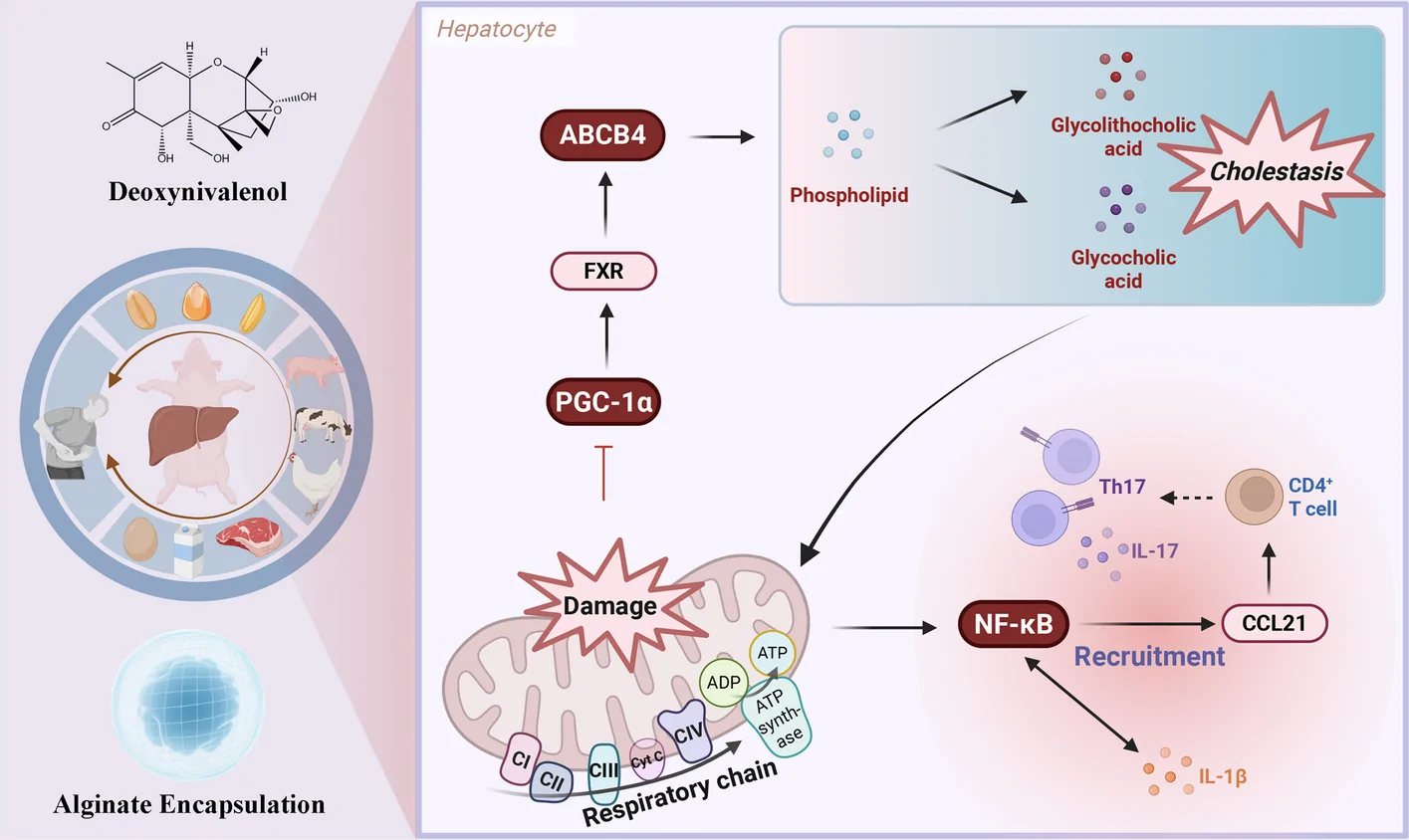

**Supplementary Information:**

The online version contains supplementary material available at https://doi.org/10.1186/s40104-026-01484-x.

## Introduction

Fungi produce natural secondary metabolites known as mycotoxins, which frequently contaminate food and animal feed [1]. Deoxynivalenol (DON), a type B trichothecene mycotoxin generated by *Fusarium graminearum* and *Fusarium culmorum*, is primarily found in cereal grains such as wheat, maize, barley, and oats [2, 3]. Although more than 500 mycotoxins have been identified, DON remains one of the most prevalent in cereal grains [4, 5]. A comprehensive global survey conducted over ten years across 100 countries found that 64% of feed ingredients and feed were contaminated with DON, with East Asia recording the highest detection rate at 84.8% [2]. According to a survey of 3,507 feed samples, the DON contamination rate in China reached 96.4% [3]. Two recent surveys showed that the maximum DON content in complete pig feed in China reached 2.26 and 4.28 mg/kg, respectively [5, 6]. Furthermore, a comprehensive review assessing the global exposure risks to DON in food over the past decade revealed that the majority of positive samples contained DON concentrations exceeding the permissible limits [7].

Common symptoms of DON exposure include vomiting, diarrhea, and food refusal [8]. Mitochondrial dysfunction, marked by reduced respiratory ability, disrupted dynamics, and excessive reactive oxygen species (ROS) generation, is considered a crucial element in DON-induced intestinal barrier damage and inflammatory responses [9–11]. Previous studies have predominantly focused on the intestinal toxicity of DON, whereas the liver, as a crucial organ for DON metabolism, has rarely been evaluated [12–14]. Notably, studies have reported that the level of DON in liver tissue is considerably greater than in other tissues following ingestion, raising growing concern regarding its potential hepatotoxicity [15, 16]. The hepatotoxic effects of DON are characterized by inflammatory cytokine release and typically involve the activation of mitogen-activated protein kinase (MAPK) and nuclear factor κB (NF-κB) signaling pathways [17, 18]. Although studies have confirmed that DON can induce hepatic inflammatory infiltration, apoptosis, and ferroptosis, the underlying mechanisms remain to be systematically elucidated [13, 14, 19, 20]. The high prevalence and concerning levels of DON contamination in grains and feed underscore the urgent need for a comprehensive understanding of the mechanisms underlying its hepatotoxicity and the development of effective detoxification strategies. Nevertheless, the remarkable chemical stability of DON complicates its elimination, posing a persistent threat to both human and animal health [21, 22].

The epoxide group at C-12,13 and the hydroxyl group at C-3 are the primary structural features responsible for the toxicity of DON [8]. Conventional physical and chemical treatment methods exhibit certain limitations, including incomplete degradation, loss of nutritional components following physical treatments, and secondary pollution caused by chemical reagents [23]. The conversion of DON into less toxic metabolites by microorganisms and enzymes constitutes a promising biological approach for the environmentally benign removal of DON [23]. Nonetheless, the intricate nature of microbial metabolism often results in an inefficient and unpredictable degradation process [23, 24]. In contrast, enzymatic detoxification of DON presents a promising alternative for its removal from cereals, feed, and food, owing to its rapid reaction rate, high efficiency, specificity, and well-defined byproducts [22, 25]. The enzymatic degradation of DON primarily proceeds through two pathways, both of which share a common intermediate. The first pathway involves C-3 epimerization, wherein DON is first oxidized to 3-keto-DON by a pyrroloquinoline-quinone (PQQ)-dependent dehydrogenase and then reduced to 3-epi-DON by a nicotinamide adenine dinucleotide phosphate (NADPH)-dependent aldo-keto reductase. In the second pathway, the same 3-keto-DON intermediate is directly converted to DOM-1 by an epoxide hydrolase [22, 26].

Gastric juice, with its extreme pH and proteases, constitutes a challenging environment that rapidly inactivates enzymes. Therefore, developing strategies to maintain enzymatic activity and achieve its precise delivery in the body is crucial. Alginate, an anionic polysaccharide primarily found in the outer cell walls of brown algae, is composed of β-D-mannuronic acid and α-L-guluronic acid [27, 28]. Due to its pH-sensitive nature, alginate can form a protective gel under acidic conditions, which subsequently swells and disintegrates upon leaving the acidic environment [28, 29]. Currently, the delivery of protein molecules using alginate hydrogel holds promise in biomedical applications [30–34]. Furthermore, the low cost and high biocompatibility of alginate underscore its potential as an enzyme encapsulation material. To our knowledge, this is the first report on the efficacy of alginate-encapsulated enzymes in degrading DON in vivo.

In this study, we systematically investigated the mechanisms underlying DON-induced liver damage using a multi-omics approach and proposed innovative strategies to mitigate the long-term health risks associated with DON exposure in animals.

## Materials and methods

### Reagents

DON was acquired from Pribolab Bioengineering Co., Ltd. (Qingdao, China), dissolved in methanol and added to feed through primary dilution to ensure homogeneous mixing. Specifically, DON was first dissolved in methanol to prepare a stock solution and then sprayed onto a basal diet (accounting for approximately 5% of the total feed). The feed was subsequently left to air-dry at room temperature for 24 h with occasional stirring to ensure complete evaporation of the methanol. Thereafter, the treated portion was thoroughly mixed with the remaining 95% of the feed. The DON-degrading enzymes (DDE; PQQ-dependent dehydrogenase DepA/NADPH-dependent aldo-keto reductase DepB, enzyme activity ≥ 5 × 10^6^ U/g), recombinantly expressed in *Escherichia coli* BL21, were acquired from Panshuo Biotechnology Co., Ltd (Hebei, China). After fermentation, the cells were harvested by centrifugation and disrupted by high‑pressure homogenization to release intracellular enzymes. The crude lysate was clarified by membrane filtration to obtain a refined enzyme solution. The DDE was encapsulated using alginate as a protective material. Specifically, the carrier (maltodextrin) and protective agents (trehalose and sucrose) were each added to deionized water and stirred to facilitate dissolution. Subsequently, the enzyme solution was added to the mixture of the carrier and protectants and gently stirred to mix uniformly. The mixture was then spray-dried to obtain a high-concentration enzyme powder, following a previously described method [35]. A 2% (w/v) alginate solution was prepared by dissolving sodium alginate in deionized water. Calcium chloride was used as the crosslinking agent to encapsulate the enzyme powder via spray-drying, according to the procedures described in prior study [36].

### Animals

Eighteen piglets (Duroc × Landrace × Yorkshire) weaned at 21 days of age were randomly assigned to three groups, each with three females and three castrated males. Specifically, pigs were divided by sex and then randomly assigned to one of three groups within each body weight (BW) stratum. The piglets were allowed to acclimatize to their living conditions for 7 d prior to the experiment. Control group (CON): basal diet (Table S1); DON group: basal diet with 2.1 mg DON/kg feed; DDE group: basal diet with 2.1 mg DON/kg feed and 20 mg DON-degrading enzymes/kg feed. DON concentration in the feed was quantified using the Chinese National Standard method [37]. The measured DON concentration in the DON and DDE groups was 2.1 mg/kg feed, while the CON group contained no detectable DON (< 0.1 mg/kg). All piglets had free access to feed and water throughout the experimental period, which lasted for 21 d. On d 14 and 21, the BW of the piglets were recorded to calculate their average daily gain (ADG). After the experiment, the piglets were euthanized following an overnight fast, and liver samples were collected and stored at −80 °C for further analysis. The content of DON in the liver was analyzed using ultra-performance liquid chromatography-tandem mass spectrometry (UPLC-MS/MS, Agilent G6470B) as previously described [38]. Simultaneously, blood was collected using coagulation accelerator tubes and centrifuged at 3,000 × *g* for 10 min to obtain serum samples for subsequent analysis. The pH of ileal and colonic digesta was measured using a calibrated digital pH meter within 30 min of collection.

### Histological analysis

Liver tissue samples were taken and promptly immersed in 4% paraformaldehyde for 48 h, then dehydrated through a gradient of ethanol (six steps, 1.5 h each), cleared in xylene (three changes, 10 min each). After paraffin embedding, the tissue was sectioned at 4 μm thickness using a microtome. Sections were then stained with hematoxylin for 3 min followed by eosin for 1 min. An optical microscope (Nikon Eclipse E100, Tokyo, Japan) was used to scan the stained slides, and images of several random fields per section were captured.

### Transmission electron microscopy (TEM)

For TEM, small liver tissue pieces (1 mm^3^) were fixed with glutaraldehyde, followed by dehydration and embedding. Following polymerization, ultrathin sections were cut at 70 nm using a Leica UC7 ultramicrotome (Leica, Wetzlar, Germany) and stained with a 2% uranium acetate saturated alcohol solution and 2.6% lead citrate. Subsequently, images were taken with a TEM (Hitachi HT7800, Tokyo, Japan).

### Transcriptomics

Transcriptomics was performed as described before [39]. In summary, liver RNA was isolated using TRIzol, and the Agilent 2100 Bioanalyzer (Agilent Technologies, CA, USA) was employed to assess RNA integrity. The pooled cDNA libraries were sequenced on an Illumina platform by Novogene Co., Ltd. (Beijing, China) [40]. According to the manufacturer's instructions, cDNA fragments of 370–420 bp were selected for PCR amplification. The PCR products were purified using AMPure XP beads for library construction, and the final libraries were sequenced on an Illumina NovaSeq 6000. Raw sequencing data were filtered to produce clean data. The reference genome index was built using HISAT2 v2.0.5, and the paired-end clean reads were aligned to the reference genome using the same software. Differential genome expression analysis was conducted using the DESeq R package. Ultimately, significant differential expression was determined by FDR‑adjusted *P* < 0.05 and |log_2_FC| > 1. Following this, KEGG enrichment analysis was conducted on these differentially expressed genes (DEGs).

### Liver damage enzymes

The levels of alanine aminotransferase (ALT), aspartate aminotransferase (AST), and gamma-glutamyl transferase (GGT) in the serum were measured using commercial kits (Biosino, Beijing, China). Meanwhile, the levels of alkaline phosphatase (ALP), cholinesterase (CHE), and lactate dehydrogenase (LDH) in the serum were quantified using ELISA kits (mlbio, Jiangsu, China). All steps in the experiment were conducted in accordance with the manufacturer's directions.

### Inflammatory cytokines

Approximately 0.1 g of liver tissue was mixed with 400 μL of saline solution and homogenized using a tissue grinder. Then, the sample was centrifuged at 3,000 × *g* for 10 min to collect the supernatant for subsequent analysis. All procedures were performed at 4 °C. Interleukin-1β (IL-1β, MM-042201), interleukin-10 (IL-10, MM-042501), interleukin-6 (IL-6, MM-041801), interleukin-17 (IL-17, MM-037701), interferon-γ (IFN-γ, MM-041201), and tumor necrosis factor-α (TNF-α, MM-038301) in the liver and serum were quantified using ELISA kits (mlbio, Jiangsu, China) according to the manufacturer’s instructions.

### Metabolomics

Untargeted metabolomics were performed as previously described [41]. In brief, the extracted liver samples were analyzed using a Vanquish UHPLC system (ThermoFisher, Germany) coupled with an Orbitrap Q Exactive™ HF mass spectrometer (ThermoFisher, Germany) [42]. Peak extraction and quantification from the data files were carried out with XCMS, aligning peaks from various samples according to retention time, mass-to-charge ratio, and other parameters. Then, metabolites were recognized using a mass deviation of 10 ppm and adduct ion information. The identified metabolites were annotated using the Kyoto Encyclopedia of Genes and Genomes (KEGG) database (https://www.genome.jp/kegg/pathway.html). The data were transformed, and principal component analysis (PCA) was conducted using MetaX. Volcano plots were created using R package ggplot2. The screening criteria for differential metabolites were VIP > 1, *P* < 0.05, and |log_2_FC| > 0.58.

### Bile acids

Approximately 0.1 g of liver tissue was mixed with 400 μL of saline solution and homogenized using a tissue grinder. The sample was then centrifuged at 3,000 × *g* for 10 min to collect the supernatant for subsequent analysis. All procedures were performed at 4 °C. Taurocholic acid (TCA, MM-92856701), glycocholic acid (GCA, MM-92823101), glycochenodeoxycholic acid (GCDCA, MM-92882901), glycolithocholic acid (GLCA, MM-92908101), and glycodeoxycholic acid (GDCA, MM-92799001) in the liver and serum were quantified using ELISA kits (mlbio, Jiangsu, China) according to the manufacturer’s instructions. The detection ranges were as follows: 15–240 pg/mL for GDCA, 16.25–260 pg/mL for GCDCA, 6.25–100 pg/mL for GLCA, 10–160 ng/mL for GCA, and 2.5–40 ng/mL for TCA. Intra‑ and inter‑assay coefficients of variation for all kits were < 15%.

### Quantitative analysis of GLCA in the liver

Liver samples were spiked with 50 μL of internal standard working solution (200 ng/mL, CDCA‑D4 and CA‑D4) and extracted with 350 μL of methanol/water (4:1, v/v). The mixture was ultrasonicated at 5 °C for 30 min, and kept at −20 °C for 30 min. After centrifugation (13,000 × *g*, 4 °C, 15 min), the supernatant was evaporated under nitrogen. The residue was reconstituted in 100 μL of 50% acetonitrile/water, ultrasonicated for 10 min, and centrifuged again. The final supernatant was used for analysis. The LC-MS/MS analysis of sample was conducted on a ExionLC AD system coupled with a QTRAP^®^6500 + mass spectrometer (SCIEX, USA). Chromatographic separation was performed on a Waters BEH C18 column (150 mm × 2.1 mm, 1.7 μm). The mobile phase consisted of solvent A (water containing 0.025% formic acid and 10 mmol/L ammonium acetate) and solvent B (acetonitrile:methanol = 9:1, v/v). The flow rate was set to 0.4 mL/min. Mass spectral data was acquired in negative ion scan mode. The optimal parameters were set as follows: Curtain Gas: 35 psi, Collision Gas: Medium, IonSpray Voltage: −4500V, Temperature: 550 °C. Peak area ratios (GLCA/internal standard) were calculated using AB Sciex OS software. The GLCA concerntration was calculated according to linear regression standard curve.

### Reverse transcription quantitative polymerase chain reaction (RT-qPCR)

According to the manufacturer’s protocol, TRIzol reagent (Aidlab, Beijing, China) was used to extract total RNA from the liver. RNA concentration was quantified by assessing the absorbance at 260 nm, and RNA purity was evaluated by determining the absorbance (A_260_/A_280_) ratio with 1.8–2.0. Total RNA was reverse transcribed into cDNA using the color reverse transcription kit (EZBioscience, USA). RT-qPCR was performed using Taq Pro Universal SYBR qPCR Master Mix (Vazyme, China) on an ABI 7500 fast RT-PCR system. Data analysis was performed with the 2^−ΔΔCt^ method, and the results were expressed as relative mRNA expression levels. β‑Actin was used as the reference gene for normalization. The stability of β‑actin across treatment groups was verified by the lack of significant variation in Ct values. The sequences of the primers used are provided in Table S2.

### Western blot

Total protein was extracted from liver tissues using RIPA lysis buffer (P0013B) containing protease and phosphatase inhibitors (P1050). A BCA protein assay kit (P0010S) was used to measure the protein concentration. Equal amounts of protein (30 μg per lane) were separated by SDS‑PAGE (P0015L) and transferred onto polyvinylidene fluoride membrane. All of the above reagents were purchased from Beyotime Biotechnology (Shanghai, China). The membranes were blocked in rapid blocking liquid (PS108P, Epizyme Biotechnology, Shanghai, China) for 20 min at room temperature, incubated with primary antibodies at 4 °C for 12 h, followed by three washes with TBST and incubated with secondary antibodies (ABclonal, AS014, 1:5,000) 37 °C for 1 h. The primary antibodies used were as follows: β-actin (ABclonal, AC026, 1:50,000), ATP-binding cassette subfamily B member 4 (ABCB4, ABclonal, A9835, 1:1,000), adenosine monophosphate-activated protein kinase (AMPK, ABclonal, A12718, 1:2,000), phosphorylated AMPK (p-AMPK, ABclonal, AP1441, 1:2,000), PPARγ coactivator-1α (PGC-1α, ABclonal, A20995, 1:1,000), optic atrophy 1 (OPA1, CST, 80471, 1:1,000), dynamin-related protein 1 (DRP1, CST, 8570, 1:1,000), vascular cell adhesion molecule 1 (VCAM1, ABclonal, A0279, 1:1,000), CC chemokine ligand 21 (CCL21, ABclonal, A1896, 1:1,000), NF-κB-p65 (ABclonal, A2547, 1:1,000), p-NF-κB-p65 (ABclonal, AP1294, 1:20,000), farnesoid X receptor (FXR, CST, 72105, 1:1,000), and bile salt export pump (BSEP, ABclonal, A8467, 1:1,000). Protein blots were detected using an enhanced chemiluminescence detection kit (Beyotime, P0018AS) and Amersham ImageQuant 800 (Cytiva, Marlborough, MA, USA). For each target protein, four biological replicates (*n* = 4 per group) were analyzed using ImageJ. The protein concentrations were normalized to that of a β-actin loading control, and the phospho-protein was normalized to the corresponding total protein concentrations. The ratios of the abundance of normalized phospho-proteins to that of normalized total proteins were calculated and plotted.

### Immunohistochemistry

Paraffin sections of the liver tissue were subjected to dehydration, antigen retrieval, and blocking of endogenous peroxidase activity. Subsequently, the sections were incubated with anti-CD4 antibody (Maixin, RMA-0620, Fuzhou, China) for 12 h at 4 °C. After washing with PBS, a secondary antibody (Abcam, ab205718) was applied and incubated at room temperature for 45 min. Next, the slides were treated with diaminobenzidine (DAB), rinsed with water, counterstained with hematoxylin, rinsed again, and then dehydrated. The images were captured using a microscope (Nikon E100, Tokyo, Japan), and quantitative analysis was conducted using ImageJ software.

### Statistical analysis

SPSS 27.0 (IBM-SPSS Inc, Chicago, IL, USA) and GraphPad Prism 8 (GraphPad Prism Inc., La Jolla, CA, USA) were utilized for statistical analyses and data visualization. One-way ANOVA with Duncan’s multiple comparison test was used for statistical evaluation as indicated. The values are presented as the mean ± SEM, and *P* < 0.05 was considered to indicate statistical significance.

## Results

### Phenotypic characteristics and liver damage enzymes

**Fig. 1:**
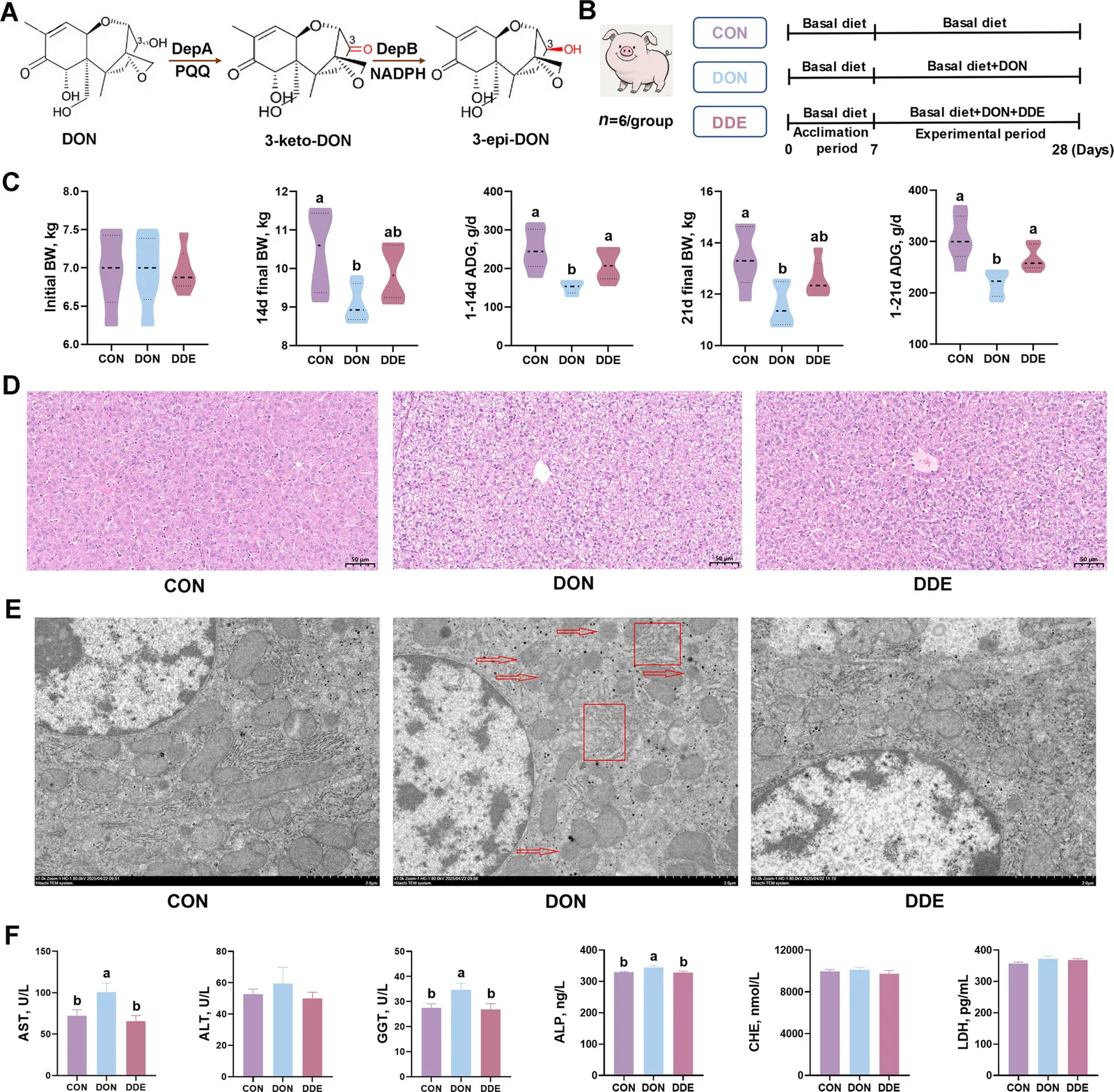
Alginate-encapsulated DDE counteracts DON-induced hepatic steatosis and mitochondrial damage in piglets (*n* = 6 per group). **A** The degradation pathway of DON in this study. **B** Schematic of experimental design. **C** Growth performance of piglets. **D** Histomorphological analysis of the liver. **E** TEM analysis of the liver. Red arrows indicate mitochondrial condensation, and the red boxes indicate slight dilation of the endoplasmic reticulum. **F** Serum levels of liver damage-associated enzymes. CON: basal diet; DON: basal diet + 2.1 mg DON/kg feed; DDE: basal diet + 2.1 mg DON/kg feed + 20 mg DDE/kg feed. Data are expressed as the mean ± SEM. ^a,b^Different superscript letters indicate significant difference at *P* < 0.05

The DDE employed in this study degrades DON via the pathway illustrated in Fig. 1A. To investigate the effects of DON on liver damage and its underlying mechanisms, we established a piglet model exposed to DON for 21 d (Fig. 1B). Meanwhile, the protective effects of dietary supplementation with alginate-encapsulated DDE against liver damage was also examined. For the pH of ileal and colonic digesta in piglets, the results showed no significant difference among the treatment groups (*P* > 0.05; Table S3). We also evaluated the growth performance of the piglets during the trial period. As shown in Fig. 1C, DON exposure significantly reduced the ADG of piglets, while dietary supplementation with DDE alleviated the DON-induced growth retardation (*P* = 0.001). Meanwhile, dietary DDE supplementation significantly reduced hepatic DON accumulation (*P* < 0.05; Fig. S1). Subsequently, we analyzed the histomorphology and ultrastructure of the liver in piglets using HE staining and TEM. Histomorphological analysis revealed that DON exposure induced qualitative evidence of hepatic steatosis, characterized by the accumulation of lipid droplets forming vacuoles within hepatocytes (Fig. 1D). TEM analysis further revealed mitochondrial condensation in hepatocytes exposed to DON, accompanied by a loss of cristae and mild dilation of the endoplasmic reticulum (Fig. 1E). Additionally, DDE supplementation alleviated DON-induced hepatic steatosis and hepatocellular damage, although mild vacuolization persisted. To further characterize the hepatic damage, we measured the levels of liver damage-related enzymes in the serum. As shown in Fig. 1F, DON exposure significantly upregulated the levels of AST, GGT, and ALP (*P* < 0.05). In contrast, compared with the DON group, the DDE group exhibited significantly reduced levels of AST, GGT, and ALP (*P* < 0.05). Collectively, these data suggest that DON exposure leads to growth retardation, hepatic steatosis, and liver damage, which were restored following DDE supplementation.

### Liver transcriptome analysis

**Fig. 2:**
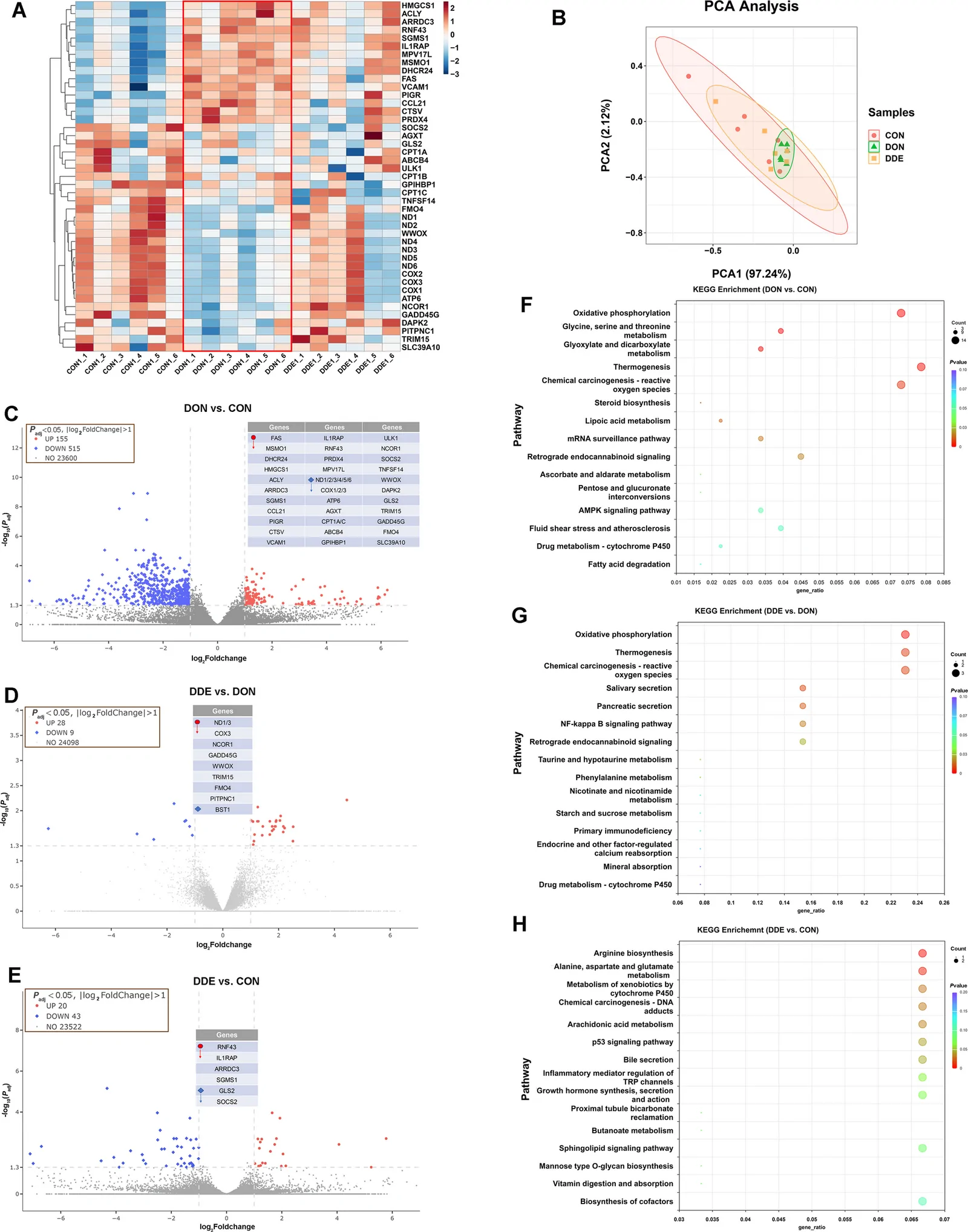
Transcriptomic profiling reveals the mechanism underlying DON-induced liver damage and the protective effect of DDE (*n* = 6 per group). **A** Clustering heat map of the genes. Blue denotes low expression and red denotes high expression. **B** Principal component analysis of liver. **C** Differentially expressed genes (DEGs) in DON vs. CON. **D** DEGs in DDE vs. DON. **E **DEGs in DDE vs. CON. *P* < 0.05, |log_2_FC| > 1; Red represents up-regulated, blue represents down-regulated. **F** KEGG pathway analysis of the differentially expressed genes in the comparisons DON vs. CON. **G** DDE vs. DON. **H** DDE vs. CON. CON: basal diet; DON: basal diet + 2.1 mg DON/kg feed; DDE: basal diet + 2.1 mg DON/kg feed + 20 mg DDE/kg feed

To further elucidate the mechanism of DON-induced liver damage and the protective effects of DDE, we conducted transcriptomic analysis. A heatmap was constructed to visualize gene expression, revealing that the gene expression profile of the DON group was distinct from those of the CON and DDE groups (Fig. 2A). PCA further revealed minimal compositional differences between the CON and DDE groups, both of which were distinctly separated from the DON group (Fig. 2B). Volcano plots were utilized to identify DEGs between the treatment groups. Specifically, we found 670 DEGs, with 155 genes up-regulated and 515 genes down-regulated between the CON and DON groups (Fig. 2C). Subsequently, we performed KEGG pathway enrichment analysis, and the top 20 enriched KEGG pathways were primarily linked to oxidative phosphorylation, glycine, serine and threonine metabolism, glyoxylate and dicarboxylate metabolism, and thermogenesis (Fig. 2F). A comparison of expressed genes between the DON and DDE groups revealed 37 DEGs, among which 28 were upregulated and 9 were downregulated (Fig. 2D). Further KEGG enrichment analysis revealed that these DEGs are primarily involved in oxidative phosphorylation and thermogenesis (Fig. 2G). Finally, a comparison between the DDE and CON groups revealed 63 DEGs, of which 20 were up-regulated and 43 were down-regulated, primarily enriched in arginine biosynthesis (Fig. 2E and H). This pattern suggests that DDE mitigates DON-induced suppression of pathways associated with oxidative phosphorylation and thermogenesis in liver tissue.

### Mitochondrial dysfunction

**Fig. 3:**
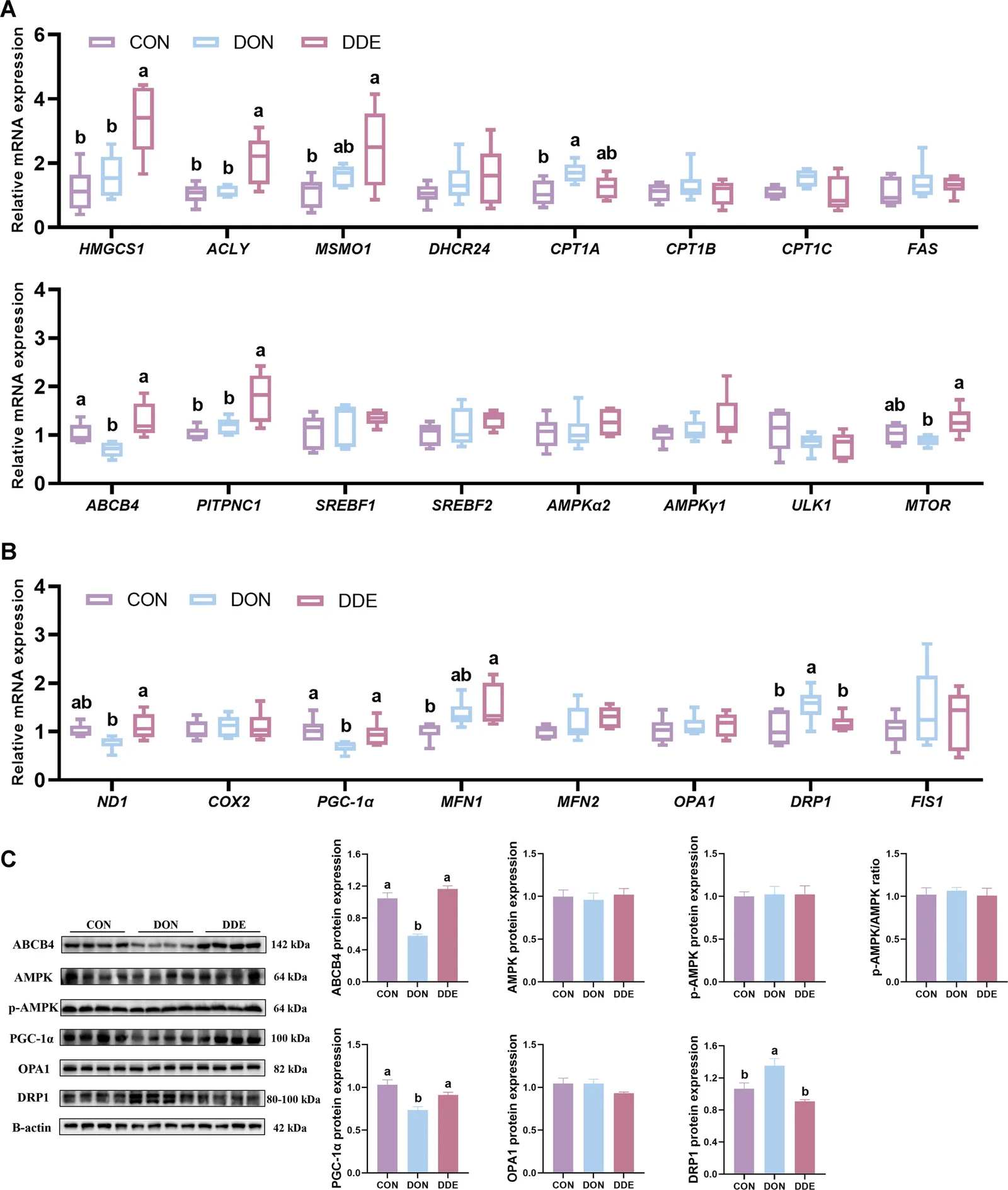
Alginate-encapsulated DDE rescues DON-induced mitochondrial dysfunction via modulation of the key factors PGC-1α, DRP1, and ABCB4 in the liver. **A** Relative mRNA expression of genes related to lipid metabolism (*n* = 6 per group). **B** Relative mRNA expression of genes related to mitochondrial function (*n* = 6 per group). **C** Western blot analysis validates the protein expression of key targets (*n* = 4 per group). CON: basal diet; DON: basal diet + 2.1 mg DON/kg feed; DDE: basal diet + 2.1 mg DON/kg feed + 20 mg DDE/kg feed. Data are expressed as the mean ± SEM. ^a,b^Different superscript letters indicate significant difference at *P* < 0.05

Liver transcriptomics revealed the enriched pathways for most DEGs were oxidative phosphorylation and thermogenesis; therefore, we further evaluated the relative mRNA and protein expression. As shown in Fig. 3A and B, DON significantly increased the relative mRNA expression of *CPT1A* and *DRP1* in the liver, while significantly reducing the relative mRNA expression of *ABCB4* and *PGC-1α* (*P* < 0.05). Moreover, compared with the DON group, DDE supplementation significantly up-regulated the relative mRNA expression of *ND1*,* HMGCS1*,* ACLY*,* ABCB4*,* PITPNC1*,* MTOR*, and *PGC-1α* (*P* < 0.05). Notably, DDE supplementation significantly decreased the relative mRNA expression of *DRP1* compared to the DON group (*P* < 0.05). As expected, Western blot analysis further confirmed that DON exposure significantly downregulated the protein expression of ABCB4 (*P* < 0.001) and PGC-1α (*P* = 0.004), while significantly upregulating *DRP1* expression (*P* = 0.004; Fig. 3B). Conversely, compared to the DON group, DDE supplementation significantly increased the protein expression of ABCB4 and PGC-1α, while significantly reducing the protein expression of DRP1 (*P* < 0.05). These data suggest that DON induced mitochondrial dysfunction, which was alleviated following DDE supplementation.

### Activated inflammatory state

**Fig. 4:**
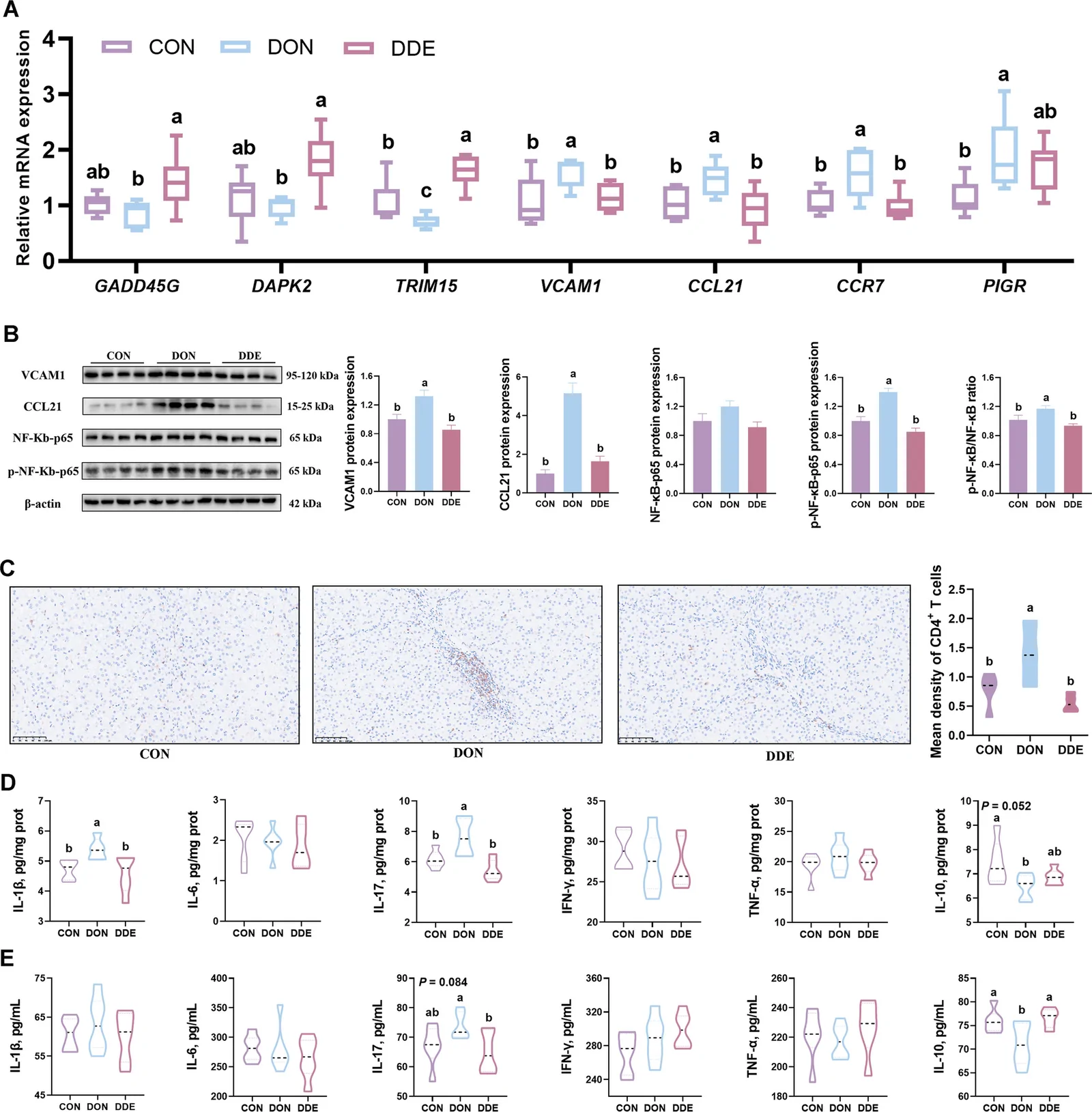
Alginate-encapsulated DDE ameliorates DON-induced hepatic inflammation by suppressing both CCL21/NF-κB pathway activation and CD4^+^ T cell recruitment. **A** Relative mRNA expression levels of inflammation-related genes in the liver (*n* = 6 per group). **B** Protein expression levels of VCAM1, CCL21, and NF-κB in the liver (*n* = 4 per group). **C** Representative immunohistochemical images and quantitative analysis of CD4^+^ T cell infiltration in the liver (*n* = 4 per group). **D** Levels of inflammatory cytokines in the liver (*n* = 6 per group). **E** Levels of inflammatory cytokines in the serum (*n* = 6 per group). CON: basal diet; DON: basal diet + 2.1 mg DON/kg feed; DDE: basal diet + 2.1 mg DON/kg feed + 20 mg DDE/kg feed. Data are expressed as the mean ± SEM. ^a–c^Different superscript letters indicate significant difference at *P* < 0.05

Based on the upregulation of inflammatory factors in the DON-treated group from the transcriptomic analysis results, we first assessed the gene and protein expression levels. Compared with the CON group, DON significantly increased the mRNA level of vascular cell adhesion molecule 1 (*VCAM1*),* CCL21*,* CCR7*, and *PIGR* (*P* < 0.05; Fig. 4A). Notably, the mRNA levels of *VCAM1*,* CCL21*,* CCR7*, and *PIGR* in the DDE group showed no significant difference compared to the CON group (*P* > 0.05). Furthermore, DDE alleviated the suppression of the relative mRNA expression of *GADD45G*,* DAPK2*, and *TRIM15* mediated by DON (*P* < 0.05). As shown in Fig. 4B, Western blot results revealed a significant upregulation in the protein expression levels of VCAM1, CCL21 and p-NF-κB-p65 in the DON group compared to the CON group (*P* < 0.05). In contrast to the DON group, the protein expression levels of VCAM1, CCL21, and p-NF-κB-p65 were significantly decreased in the DDE group (*P* < 0.05). Given the role of the chemokine CCL21 in T cell recruitment, we further analyzed the expression of CD4 in the liver using immunohistochemistry. As expected, DON exposure induced a significant upregulation of CD4 expression (*P* < 0.05), while no significant difference was observed compared to the DDE-treated pigs (*P* > 0.05; Fig. 4C). Further analysis of inflammatory cytokines revealed that DON exposure upregulated the levels of IL-1β and IL-17 in the liver, while downregulating the level of IL-10 in the serum (*P* < 0.05) (Fig. 4D and E). Notably, DDE supplementation down-regulated the levels of IL-1β and IL-17 in the liver while up-regulated the level of IL-10 in the serum (*P* < 0.05). These data indicate that DON exposure facilitates the recruitment of T cells via the chemokine CCL21 as part of the inflammatory response, whereas DDE supplementation alleviates inflammatory activation.

### Liver metabolites analysis

**Fig. 5:**
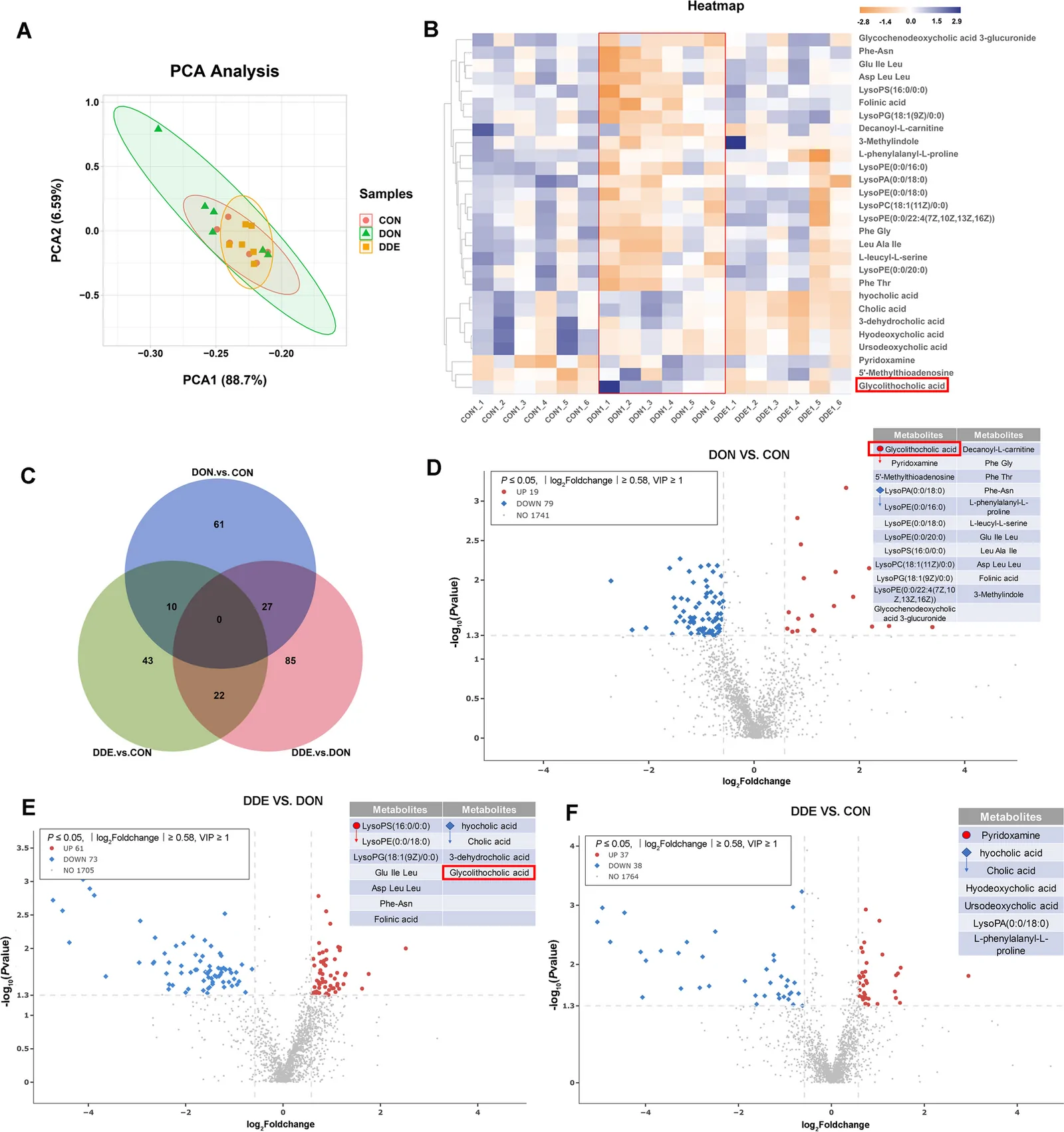
Hepatic metabolic profile in response to DON exposure and DDE intervention (*n* = 6 per group). **A** Principal component analysis of metabolic profiles in the liver. **B** Heatmap visualizing the abundance patterns of metabolites. Blue denotes high abundance and red denotes low abundance. **C** Venn diagram illustrating the number of overlapping differential metabolites. **D** Differential metabolites between DON and CON. **E** Differential metabolites between DDE and DON. **F** Differential metabolites between DDE and CON. CON: basal diet; DON: basal diet + 2.1 mg DON/kg feed; DDE: basal diet + 2.1 mg DON/kg feed + 20 mg DDE/kg feed

**Fig. 6:**
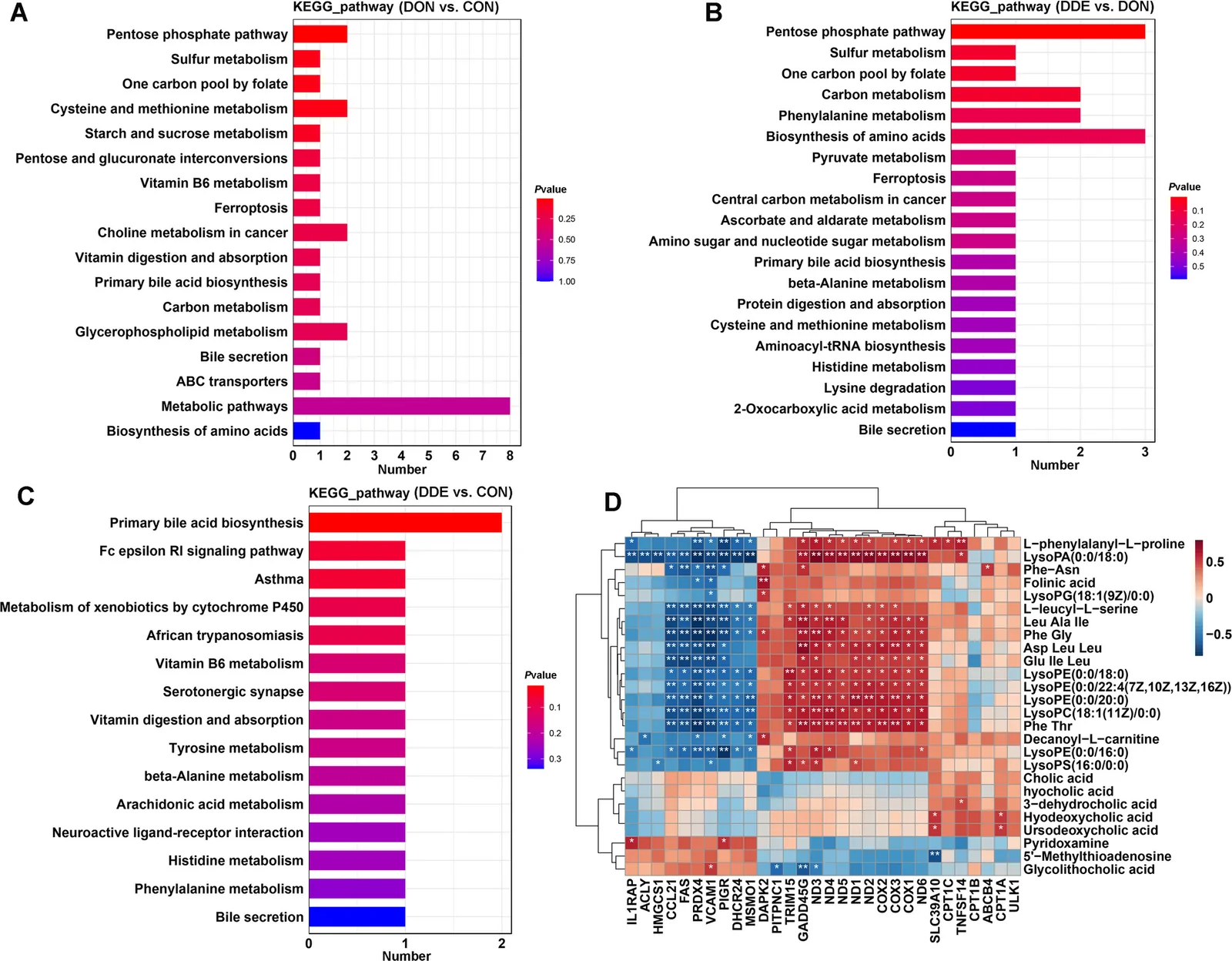
Pathway enrichment and correlation analysis of differential metabolites (*n* = 6 per group). **A** KEGG pathway enrichment analysis of differential metabolites in the DON vs. CON comparison. **B** Differential metabolites in DDE vs. DON. **C** Differential metabolites in DDE vs. CON. **D** Heatmap of Spearman's correlation coefficients between differentially expressed genes and altered metabolites. Red indicates a positive correlation, and blue indicates a negative correlation. CON: basal diet; DON: basal diet + 2.1 mg DON/kg feed; DDE: basal diet + 2.1 mg DON/kg feed + 20 mg DDE/kg feed

Based on the transcriptomic findings regarding the regulation of mitochondrial respiration and lipid metabolism by DON, we further employed untargeted metabolomics to analyze the differences in metabolite composition among the groups. PCA plot revealed a distinct separation between the DON group and the CON or DDE-treated groups, despite some partial overlap (Fig. 5A). Consistent with the PCA, the heatmap showed that the metabolite profile of the DON group differed from those of the CON and DDE groups (Fig. 5B). A Venn diagram illustrated the overlap of altered metabolites among the comparisons, showing that the DON vs. CON and DDE vs. DON comparisons shared the greatest number of common metabolites (27) (Fig. 5C). To further characterize these alterations, volcano plots were generated to visualize the differential metabolites. An analysis of metabolites in liver revealed 98 differentially abundant metabolites between the CON and DON groups, with 79 metabolites being downregulated and 19 metabolites upregulated (Fig. 5D). Specifically, the levels of various lysophospholipids and small peptides were downregulated, while the levels of GLCA were upregulated. Moreover, 61 metabolites were increased and 73 metabolites were decreased in the DDE group compared to the DON group (Fig. 5E). Interestingly, DDE supplementation increased the levels of various lysophospholipids and small peptides, while reducing the levels of various bile acids. However, a comparison between the DDE and CON groups still revealed reduced levels of various bile acids, whereas only pyridoxamine exhibited a significant increase (Fig. 5F). Pathway enrichment analysis using the KEGG database was performed on these differential metabolites. This revealed significant enrichment of metabolites associated with the pentose phosphate pathway in the DON vs. CON comparison (Fig. 6A). Notably, the pentose phosphate pathway and biosynthesis of amino acids were enriched in the DDE vs. DON comparison (Fig. 6B). In contrast to the comparison between the DON and CON groups, these pathways showed significant upregulation. Conversely, no enrichment of the pentose phosphate pathway was observed in the DDE vs. CON comparison; however, enrichment of the primary bile acid biosynthesis was detected (Fig. 6C).

### Potential correlation between the transcript profiles and metabolites

We created a correlation matrix using Spearman's correlation analysis and produced heatmaps to clarify the functional relationships between DEGs and metabolites (Fig. 6D). In the heatmap, red squares represent positive correlations, while blue squares represent negative correlations. The inflammation-related genes *CCL21*, *VCAM1*, and *PIGR* were significantly negatively correlated with various lysophospholipids (lysoPA, lysoPE, and lysoPC) and small peptides (*P* < 0.05). Meanwhile, cholesterol synthesis-related genes *DHCR24* and *MSMO1* were also significantly negatively correlated with these lysophospholipids and small peptides (*P* < 0.05). In contrast, mitochondrial genes (*ND1*,* ND2*,* ND3*,* ND4*,* ND5*,* ND6*,* COX1*,* COX2*,* COX3*), along with *TRIM15* and *GADD45G*, demonstrated significant positive correlations with lysoPA, lysoPE, and lysoPC (*P* < 0.05). Notably, the metabolite GLCA was significantly negatively correlated with the genes *PITPNC1* and *GADD45G*, while exhibiting a significant positive correlation with *VCAM1* (*P* < 0.05). These results suggest that exposure to DON causes notable changes in gene expression, which are linked to alterations in metabolite profiles.

### Bile acid metabolism

**Fig. 7:**
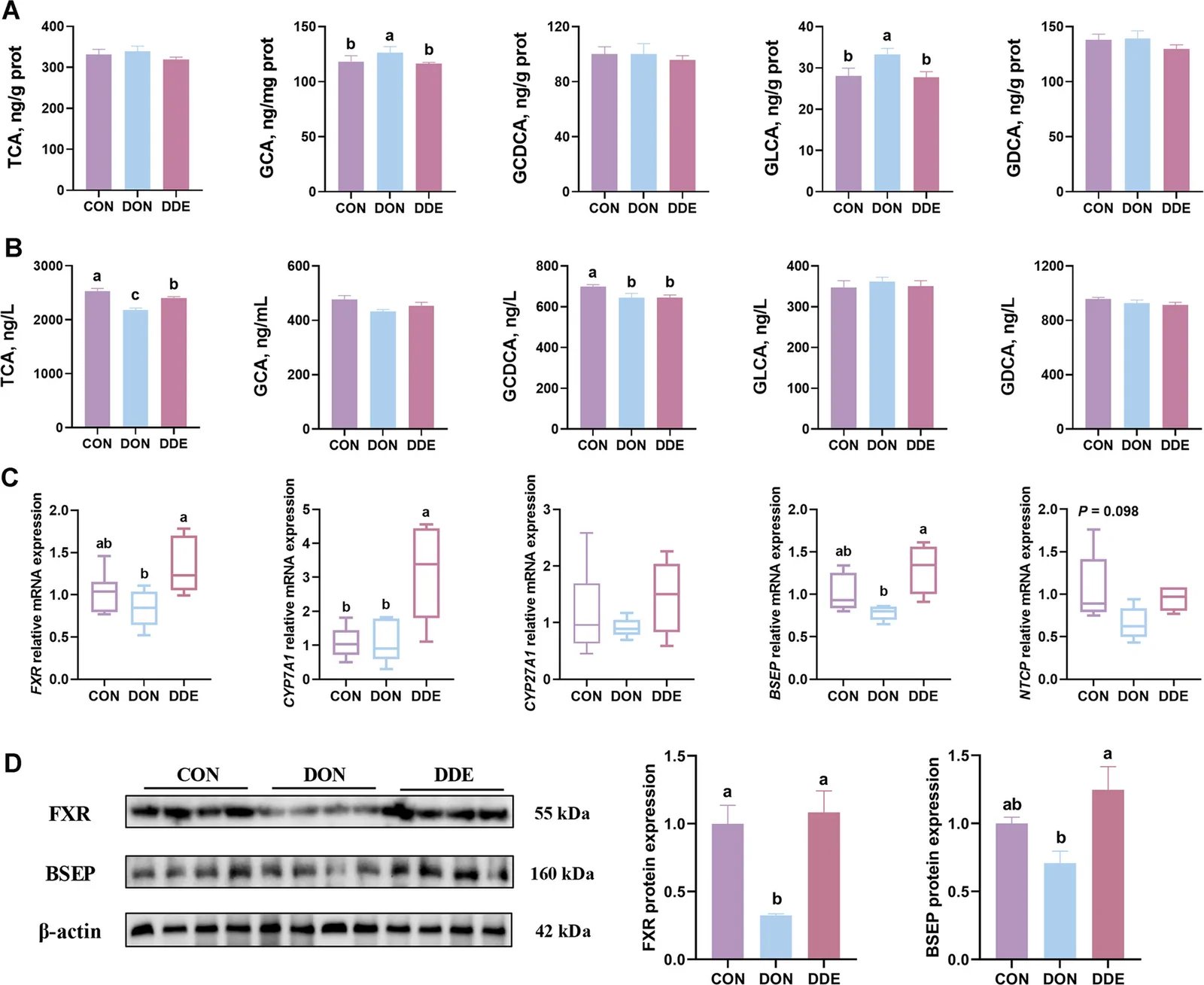
Alginate-encapsulated DDE reversed DON-induced accumulation of GLCA and GCA in the liver (*n* = 6 per group). **A** Levels of bile acids in the liver. **B** Levels of bile acids in the serum. **C** Relative mRNA expression of bile acid metabolism-related genes. **D** Representative Western blot images and quantitative analysis of FXR and BSEP protein expression (*n* = 4 per group). CON: basal diet; DON: basal diet + 2.1 mg DON/kg feed; DDE: basal diet + 2.1 mg DON/kg feed + 20 mg DDE/kg feed. Data are expressed as the mean ± SEM. ^a,b^Different superscript letters indicate significant difference at *P* < 0.05

Given the differences in various lysophospholipids and bile acids identified in the metabolomic analysis, we further measured the levels of bile acids and the activation status of related signaling pathways. As shown in Fig. 7A, compared to the CON group, DON exposure significantly increased the levels of GCA (*P* = 0.030) and GLCA (*P* = 0.049) in the liver. In contrast, DDE treatment significantly downregulated the levels of these bile acids compared to the DON group (*P* < 0.05). The ELISA‑measured GLCA levels were confirmed by LC‑MS/MS analysis (Fig. S2). In serum, DON exposure significantly decreased the levels of TCA and GCDCA compared with the CON group, while supplementation with DDE increased the levels of TCA (*P* < 0.05) (Fig. 7B). Furthermore, we assessed the activation status of key factors in the bile acid synthesis and metabolism pathways (Fig. 7C and D). The results indicated that DON reduced the protein expression of FXR (*P* = 0.003). Meanwhile, compared with the DON group, DDE treatment significantly increased both the gene and protein expression levels of FXR and BSEP (*P* = 0.026). These results indicate that DDE effectively alleviates DON-induced bile acid metabolic dysfunction in the liver.

## Discussion

DON is primarily found in cereals, including wheat, maize, barley, and oats, and poses a continuous threat to animal growth and human health [2]. According to a global survey, 64% of cereal samples from 100 different countries showed DON contamination [3]. In China, more than 96.4% of feed samples contained DON, with concentrations as high as 3.7 mg/kg [3]. To date, research on the toxicity of DON has largely focused on the jejunum. With in-depth investigations into the mechanisms underlying DON toxicity, the liver has gradually gained attention as a potentially important target organ [14]. Given that the liver is the primary site of DON metabolism in both humans and animals, it is imperative to explore the mechanisms of DON-induced hepatotoxicity and develop effective mitigation strategies. In this study, we established a 21-d DON exposure model in piglets. The results indicated that DON exposure induced hepatic steatosis and mitochondrial dysfunction. Furthermore, by suppressing the expression of the critical phospholipid transporter ABCB4, it promotes the accumulation of GLCA, thereby exacerbating liver damage. Enzymes are characterized by efficiency and specificity and can target the DON for degradation, but their widespread application still faces the challenge of instability within physiological environments [43]. In this study, we employed pH-dependent alginate encapsulation to prevent enzymatic degradation in gastric acid. Remarkably, the alginate-encapsulated DDE rescued DON-induced liver damage and growth retardation in piglets.

In mycotoxin exposure trials, growth performance is regarded as the most intuitive phenotypic characteristic. As expected, DON exposure induced severe growth retardation in piglets. Considering that the liver serves as the principal organ responsible for detoxification, liver damage induced by DON exposure may be a critical factor contributing to this growth retardation. A recent study has demonstrated that DON induces hepatic vacuolation in mice, accompanied by neutrophil infiltration [17]. Therefore, we first assessed the morphology and ultrastructure of liver tissue. The results revealed that the livers of DON-exposed pigs exhibited marked steatosis and vacuolization, indicating that DON exposure induced liver damage and abnormal lipid metabolism. Meanwhile, in addition to mild endoplasmic reticulum dilation, TEM analysis also showed pronounced mitochondrial condensation, accompanied by the loss of cristae. Mitochondrial condensation are manifestations of hepatocyte apoptosis, suggesting that DON exposure causes severe damage to liver mitochondria. Remarkably, DDE supplementation effectively alleviated the DON-induced growth retardation and liver damage, characterized by the absence of vacuolization as well as mitochondrial condensation. To further corroborate the above findings, we measured the levels of relevant enzymes in the serum. Consistently, AST, GGT, and ALP were significantly elevated in DON-treated pigs. Interestingly, no significant difference in ALT was observed. The liver is the primary source of AST, which is predominantly located in the mitochondria of hepatocytes [44]. In contrast, ALT is mainly found in the cytoplasm of hepatocytes [44]. These findings further support that mitochondria are the primary target of DON-induced hepatocyte damage. It is noteworthy that GGT and ALP are primarily derived from the liver, and their simultaneous elevation typically signifies cholestasis [45]. These findings suggest that the primary manifestations of DON-induced hepatic injury include mitochondrial impairment and cholestatic changes. In comparison, no upregulation of the aforementioned enzymes was observed in the serum of pigs treated with DDE, preliminarily indicating the in vivo efficacy of this strategy.

To further investigate the mechanism by which DON exposure induces liver damage and the mitigating effect of DDE, we obtained gene expression profiles from the livers of piglets. We found that the gene expression profile of DON-exposed group was severely displaced compared with the CON group. This was manifested by the differential expression of multiple genes involved in several key pathways, including oxidative phosphorylation, glyoxylate and dicarboxylate metabolism, glycine, serine and threonine metabolism, and thermogenesis. Specifically, DON exposure decreased the expression of ND1/2/3/4/5/6, COX1/2/3, ATP6, CPT1A/C, and ABCB4, and AGXT, whereas DDE treatment resulted in higher expression levels. The NADH dehydrogenase subunits, cytochrome c oxidase subunits, and ATP6 are all encoded by mitochondrial DNA and are collectively involved in electron transport and thermogenesis within the mitochondrial respiratory chain [46]. It should be pointed out that the downregulation of the above-mentioned genes indicates severely impaired mitochondrial function accompanied by an energy crisis, which is consistent with the analysis of ultrastructural and liver damage enzymes. Therefore, we further determined the activation status of key targets involved in the regulation of cellular energy metabolism. As critical sensors and regulators of energy, AMPK and PGC-1α are typically activated under conditions of energy stress [47, 48]. Interestingly, we did not observe an upregulation in the expression of AMPK and PGC-1α. Conversely, DON exposure suppressed PGC-1α expression, which may result from mitochondrial impairment and inflammatory activation [49]. Therefore, we analyzed mitochondrial dynamics, which is essential for maintaining normal mitochondrial function. The upregulation of Drp1 gene and protein expression, responsible for regulating mitochondrial fission, further supports the conclusion that DON exposure leads to mitochondrial dysfunction and energy metabolism disorders [50]. Notably, DDE effectively reversed the mitochondrial damage and energy crisis induced by DON, as evidenced by the upregulation of ND1, ND3, COX3, and PGC-1α expression, along with the inhibition of Drp1 activation.

In addition to the mitochondrial dysfunction, upregulation of various inflammation-related factors was also observed in the liver transcriptome analysis. CCL21 and VCAM1, as chemokines and adhesion molecules respectively, are both primarily regulated by the transcription factor NF-κB [51, 52]. Therefore, NF-κB may rapidly respond to DON-induced ribotoxic stress to initiate the transcription of pro-inflammatory cytokines, chemokines, and inflammatory mediators [8, 53]. Further protein expression analysis confirmed that DON mediates the upregulation of CCL21 and VCAM1 by activating NF-κB. It should be noted that CCL21 primarily exerts its anti-inflammatory effects by recruiting naive T cells through binding to the receptor CCR7. In this study, the upregulation of CCL21 and VCAM1 induced by DON may contribute to immune cell infiltration and the amplification of inflammatory responses at the site of inflammation. Given this, immunohistochemistry and ELISA were further employed to evaluate T cell accumulation and inflammatory cytokine levels in the liver. As expected, DON exposure resulted in the upregulation of CD4 expression. NF-κB-driven expression of IL-1β and chemokines recruits immune cells, thereby further exacerbating the inflammatory response, which is consistent with the observed upregulation of IL1RAP in DON-exposed pigs [54]. IL1RAP, as a co-receptor, serves as a key amplifier of the IL-1 signaling pathway [55]. Further inflammatory cytokine assays revealed that DON induced upregulation of IL-1β and IL-17 in the liver. These results suggest that DON, through NF-κB-driven expression of IL-1β and CCL21, recruits T cells and promotes their differentiation into the pro-inflammatory Th17 subset, thereby amplifying the inflammatory response. Meanwhile, DDE treatment suppressed the expression of inflammatory factors, chemokines, and adhesion molecules induced by DON, as well as the enrichment of CD4^+^ T cells. Notably, compared to the DON group, DDE upregulated the expression of GADD45G and DAPK2. GADD45G and DAPK2 cooperatively induce apoptosis in damaged cells to maintain tissue homeostasis, while loss of DAPK2 triggers activation of NF-κB [56, 57]. Overall, DDE treatment effectively reversed the DON-induced NF-κB-driven Th17 immune response.

Given the previous speculation regarding cholestasis, we further employed metabolomics to evaluate metabolic differences in the livers of piglets among groups. The results indicated that the metabolite profile of DON-treated pigs was distinguished from that of CON and DDE-treated pigs, characterized by the upregulation of GLCA and the downregulation of various lysophospholipids. Lysophospholipids, as a unique subclass of phospholipids, are generated by the hydrolysis of phospholipids by phospholipases [58]. ABCB4, situated on the canalicular membrane of hepatocytes, protects the liver against the toxic effects of hydrophobic bile acids by transporting phospholipids for conjugation with bile acids [59, 60]. Consistent with the transcriptomic findings, further RT-qPCR and Western blot analyses indicated that DON markedly suppressed ABCB4 expression. This supports our hypothesis that DON inhibits the transport of phospholipids by downregulating ABCB4 expression, thereby leading to a reduction in lysophospholipid levels. In addition to protecting hepatocytes from bile acid toxicity, the formation of mixed micelles by phospholipids and bile acids is also a critical link in bile acid metabolism [61]. In this experiment, the reduction in phospholipid levels might cause bile acid accumulation in the liver. Therefore, we further determined the levels of various bile acids in the liver. As expected, DON exposure resulted in a significant accumulation of GCA and GLCA in the liver. Notably, alginate-encapsulated DDE effectively alleviated DON-induced cholestasis, which was characterized by the restoration of lysophospholipids and ABCB4, and the reduction of GLCA. As an important primary bile acid in the liver, GCA exhibited no significant hepatotoxicity at physiological concentrations. In contrast, GLCA, as a hydrophobic bile acid, can dissolve cell membranes and mitochondrial membranes upon its accumulation, leading to the leakage of cellular contents and disruption of energy metabolism, ultimately resulting in hepatocyte necrosis or apoptosis [62, 63]. Our findings indicate that the accumulation of GLCA, mediated by impaired phospholipid transport, was the principal factor contributing to mitochondrial dysfunction and inflammatory responses, as supported by correlation analysis. Nevertheless, the mechanism by which DON exposure leads to the downregulation of the ABCB4 transporter remains unclear. Previous studies have indicated that PGC-1α, as a coactivator of FXR, can regulate the expression of downstream targets through FXR, which may account for the observed downregulation of ABCB4 [64, 65]. Subsequent analyses of FXR expression corroborated our hypothesis, revealing that FXR expression was diminished in pigs treated with DON. These data suggest that DON-induced mitochondrial dysfunction suppresses the FXR-ABCB4 pathway by impairing PGC-1α-mediated activation. Furthermore, the inhibition of ABCB4-mediated phospholipid transport leads to the accumulation of GLCA, thereby exacerbating hepatic mitochondrial damage and inflammatory responses. Simultaneously, a decrease in the levels of various bile acids was observed in the serum, further supporting the impaired bile acid metabolism due to reduced phospholipid levels. Additionally, DON-induced ileal damage leading to impaired bile acid reabsorption may also contribute to the decrease in serum bile acids [61]. Concurrently, our results demonstrated that DDE supplementation counteracted the DON-induced inhibition of bile acid metabolism and the mitochondrial function.

Several limitations of this study should be acknowledged. First, individual feed intake was not recorded. Second, the sample size per group (*n* = 6) is relatively small, which precludes robust sex‑specific analysis. Third, the experimental design did not include a control group receiving the alginate-encapsulated enzymes or unencapsulated enzymes. Fourth, direct quantification of DON degradation products in biological samples was not performed; thus, the proposed in vivo degradation pathway remains to be conclusively verified. Future studies with larger sample sizes, individual feed intake monitoring, appropriate carrier controls, and quantitative analysis of DON metabolites are warranted to further substantiate the conclusions.

## Conclusions

In conclusion, DON exposure results in growth retardation in piglets and induces mitochondrial damage in hepatocytes, characterized by abnormal mitochondrial morphology and elevated levels of mitochondrial marker enzymes. Furthermore, DON exposure also suppresses the expression of FXR and ABCB4. As a critical component of bile acid metabolism, ABCB4-mediated GLCA accumulation may be an important factor in causing mitochondrial dysfunction and the Th17 immune response. However, further studies are required to clarify the potential mechanism of DON-induced hepatotoxicity. Leveraging the contraction properties of alginate under acidic conditions, we demonstrate that the encapsulated DDE formulation effectively rescues DON-induced hepatic mitochondrial damage, cholestasis, and Th17 immune response in vivo. This study suggests a potential ABCB4-dependent mechanism underlying DON-induced liver damage and provides a novel strategy for the in vivo elimination of foodborne DON.

## Supplementary Information

Additional file 1: Table S1. The compositions and ingredients of the diet. Table S2. The qRT-PCR primer sequences used in this study. Table S3. Effects of DON and DDE on the pH of ileal and colonic digesta in piglets. Fig. S1 DON content in the liver. Fig. S2 The concentration of GLCA in the liver was determined using LC‑MS/MS.

Additional file 2: Fig. S1. Gels and blots images.

Additional file 3. Differentially expressed genes and metabolites in the liver.

## Data Availability

The data that support the findings of this study are available from the corresponding author upon reasonable request.

## Abbreviations

ABCB4: ATP-binding cassette subfamily B member 4
ACLY: ATP-citrate lyase
AMPK: AMP-activated protein kinase
BSEP: Bile salt export pump
CCL21: C-C motif chemokine ligand 21
CCR7: C-C motif chemokine receptor 7
CPT1A: Carnitine palmitoyltransferase 1A
CPT1B: Carnitine palmitoyltransferase 1B
CPT1C: Carnitine palmitoyltransferase 1C
COX: Cyclooxygenase
CYP7A1: Cholesterol 7α-hydroxylase
CYP27A1: Sterol 27-hydroxylase
DAPK2: Death-associated protein kinase 2
DON: Deoxynivalenol
DDE: DON-degrading enzymes
DHCR24: 24-Dehydrocholesterol reductase
DRP1: Dynamin-related protein 1
FAS: Fatty acid synthase
FIS1: Fission protein 1
FXR: Farnesoid X receptor
GADD45G: Growth arrest and DNA damage inducible gamma
HMGCS1: 3-Hydroxy-3-methylglutaryl-CoA synthase 1
MFN1: Mitofusin 1
MFN2: Mitofusin 2
mTOR: Mammalian target of rapamycin
MSMO1: Methylsterol monooxygenase 1
ND1: NADH dehydrogenase subunit 1
NTCP: Sodium taurocholate cotransporting polypeptide
ULK1: Unc-51 like autophagy activating kinase 1
OPA1: Optic atrophy 1
PGC-1α: PPARγ coactivator-1α
PITPNC1: Phosphatidylinositol transfer protein cytoplasmic 1
PIGR: Polymeric immunoglobulin receptor
SREBF1: Sterol regulatory element binding transcription factor 1
SREBF2: Sterol regulatory element binding transcription factor 2
TRIM15: Tripartite motif containing 15
VCAM1: Vascular cell adhesion molecule 1
