# Supplementary material for: Alginate-encapsulated enzymes mitigate deoxynivalenol-induced hepatic mitochondrial dysfunction and glycolithocholic acid accumulation in piglets
Source: J Anim Sci Biotechnol. 2026 Sep 5;17:170. doi: 10.1186/s40104-026-01484-x (PMC13545767; doi:10.1186/s40104-026-01484-x)
Supplement: Supplementary file 1 [file 40104_2026_1484_MOESM1_ESM.docx]

**Table S1** The compositions and ingredients of the diet

| **Ingredients** | **Content** |
| --- | --- |
| Corn | 54.89 |
| Soybean meal | 8.00 |
| Enzymatically hydrolyzed soybean meal | 6.50 |
| Extruded soybean | 12.00 |
| Fish meal | 3.00 |
| Whey powder | 10.00 |
| Soybean oil | 1.30 |
| Dicalcium phosphate | 0.70 |
| Limestone | 0.75 |
| NaCl | 0.30 |
| L-Lysine | 0.55 |
| DL-Methionine | 0.23 |
| L-Threonine | 0.23 |
| L-Tryptophan | 0.05 |
| Premix^1^ | 1.50 |
| Total | 100.0 |
| Nutrient levels^2^ |  |
| Metabolizable energy, kcal/kg | 3433 |
| Crude fat | 2.25 |
| Crude protein | 18.53 |
| SID Lysine | 1.25 |
| SID Methionine | 0.49 |
| SID Threonine | 0.77 |
| SID Tryptophan | 0.23 |
| Calcium | 0.69 |
| Total phosphorus | 0.55 |
| Available phosphorus | 0.36 |

^1^ Provided the following per kilogram of diet: Fe, 120 mg; Cu, 16 mg; Mn, 70 mg; Zn, 80 mg; Se, 0.48 mg; I, 0.7 mg; vitamin A, 16, 000 IU; vitamin D, 1, 0000 IU; vitamin E, 50 mg; vitamin K_3_, 5 mg; vitamin B_1_, 3.75 mg; vitamin B_2_, 12.5 mg; vitamin B_6_, 7.5 mg; vitamin B_12_, 0.05 mg; niacin, 50 mg; d-pantothenic acid, 25 mg; d-biotin, 0.25 mg; and folate, 2.5 mg

^2^ Nutrient levels were calculated values

**Table S2** The qRT-PCR primer sequences used in this study

| **Gene** | **Primer sequence (5´→3´)** | **Accession number** |
| --- | --- | --- |
| β-actin | F: GGCACCACACCTTCTACAACGAG | XM_003124280.5 |
|  | R: TCATCTTCTCACGGTTGGCTTTGG |  |
| ABCB4 | F: AATGTCCAGTGGCTGAGAGC | XM_013989596.2 |
|  | R: CGATGTTCTCGGCAATGCTG |  |
| ACLY | F: TCCAGTCCGTGTACGAGGAT | NM_001257276.1 |
|  | R: GCGTAGATGAGCTCCTGTCC |  |
| AMPKα1 | F: CGACAGCCGAGAAGCAGAAAC | NM_001167633.1 |
|  | R: ATGTTTGCCAACCTTCACTTTGC |  |
| AMPKγ1 | F: CCACAGGCTGCCAGTTATTGAC | NM_001001642.2 |
|  | R: GCTTGAGGAACTTGAGGATGCG |  |
| BSEP | F: TTTCATTCAGCGCCTGACCA | XM_003133457.5 |
|  | R: ACTCCAATGAGAGGGCTGAC |  |
| CCL21 | F: CCAAGGCAGTGATGGAGGAG | NM_001005151.1 |
|  | R: CTTCCGGTAGCTGCGTACAA |  |
| CCR7 | F: ATGGACCTGGGGAAGCCTAT | NM_001001532.3 |
|  | R: CTTAAAGGTCCGCACGTCCT |  |
| CPT1A | F: CAAGATAGCGGCCGAAAAGC | NM_001129805.2 |
|  | R: GATAATCGCCACGGCTCAGA |  |
| CPT1B | F: GGGCTGGTCAATCACATCCA | NM_001007191.1 |
|  | R: TCCAACCGTGGTAGGAAAGC |  |
| CPT1C | F: TGCTTTCAGCTGGGCTACTC | XM_021094746.1 |
|  | R: AGACGTGGCAGTCGATGTTT |  |
| COX2 | F: CTTGCTGTTCCAACCCATGC | NM_214321.1 |
|  | R: AGAATCCTGTTCGGGTGCAG |  |
| CYP7A1 | F: GAAAGAGAGACCACATCTCGG | NM_001005352.3 |
|  | R: GAATGGTGTTGGCTTGCGAT |  |
| CYP27A1 | F: CACGACATCCAACACGCTGAC | NM_001243304.1 |
|  | R: CCATGCCCACCACCTCCTC |  |
| DAPK2 | F: AGCTGGCCAAGGACTTCATC | NM_001243563.1 |
|  | R: CAGGTTGACCACAGACTCCC |  |
| DHCR24 | F: ACAAACCAAAAGGGCCTGGA | NM_001243354.1 |
|  | R: TCAGGAGGGAGATCTTGGGG |  |
| DRP1 | F: TAAACCGAAGCCAGAAGGACA | XM_021069512.1 |
|  | R: AAGTGGCGATAGGAAGGGTG |  |
| FAS | F: TGATGCCCAAGTGACTGACC | NM_213839.1 |
|  | R: CAGCATGTTTCCGTTTGCCA |  |
| FIS1 | F: CAGACAGAGCCACAGAACAACCAG | XM_021086263.1 |
|  | R: CAAGTCCAATGAGTCCAGCCAGTC |  |
| FXR | F: TTTGTGTCGTTTGCGGAGAC | NM_001287412.1 |
|  | R: GTTGCCCCCATTTTTACACTTG |  |
| GADD45G | F: GAGTCCGCCAAAGTCCTGAA | NM_001185129.1 |
|  | R: AGCAGAACGCCTGGATCAAA |  |
| HMGCS1 | F: TCCTTTGAACGCAGAAGCCT | NM_001252215.1 |
|  | R: GTCTGTGCAGAAGCCCATCT |  |
| MFN1 | F: TGGACTTTATCCGAAACCAGATGAACC | NM_001315732.1 |
|  | R: AACCTTATTTGCCACCTCCTCTGTAAC |  |
| MFN2 | F: CCACACCACCAACTGCTTCCTG | XM_021095348.1 |
|  | R: TCTTGACGCTCCTCTTCTCCTCTG |  |
| MTOR | F: AGCCTTCGTCTATGACCCCT | XM_003127584.6 |
|  | R: CGGCAGAGTAGGAATCCGTC |  |
| MSMO1 | F: TAGTTCGGCATCCTTGGCTG | NM_213752.1 |
|  | R: CCCACGTTGCAATCTGGAAC |  |
| ND1 | F: ACCCTAGCAGAAACCAACCG | GQ338984.1 |
|  | R: TCGTGGGATGCTCCTAGGAA |  |
| NTCP | F: ACTTTCGGAAACCTAAGGGACT | XM_001927695.5 |
|  | R: AAGAGCTTGCCCAGTGCAAAG |  |
| ULK1 | F: GCCGGCTCACTGATCTACTC | XM_021062868.1 |
|  | R: TGCGATCTCCATGGGCTTTT |  |
| OPA1 | F: ACAGAGGATGGTGCTTGTTGACTTAC | XM_021070063.1 |
|  | R: ACACAGTATGATGGCGTTGGGATTC |  |
| PGC-1α | F: GGCAGAGGCAGATGTGTTCTTCC | NM_213963.2 |
|  | R: CGGCTCGGATTTCCTGGTCTTG |  |
| PITPNC1 | F: GGGGACAGTTAAGGGAAGGC | NM_001143722.1 |
|  | R: CATCAACCCAGGCAAAAGCC |  |
| PIGR | F: GTCTACGTGGCAGTGGAACA | NM_214159.1 |
|  | R: CACTGCTCTGTCCTCTGCAA |  |
| SREBF1 | F: TGCTGGCGGAGGTCTATGTGG | NM_214157.1 |
|  | R: CAGGAAGAAGCGGGTCAGAAAGTG |  |
| SREBF2 | F: CTGGTACGCTGGCTGCTCAAG | XM_021091444.1 |
|  | R: CGCTGCTCTTGGCTTCATCCTC |  |
| TRIM15 | F: AGGAGCTGGAGGAGAAGTGT | NM_001123208.1 |
|  | R: AAGTCTTCGTCTCGCACCTG |  |
| VCAM1 | F: CACAGTCTCCTGCGGGAATT | NM_213891.1 |
|  | R: TCAACCGGGTAAACGTCAGG |  |

ABCB4, ATP-binding cassette subfamily B member 4; ACLY, ATP-citrate lyase; AMPK, AMP-activated protein kinase; BSEP, bile salt export pump; CCL21, C-C motif chemokine ligand 21; CCR7, C-C motif chemokine receptor 7; CPT1A, carnitine palmitoyltransferase 1A; CPT1B, carnitine palmitoyltransferase 1B; CPT1C, carnitine palmitoyltransferase 1C; COX2, cyclooxygenase-2; CYP7A1, cholesterol 7α-hydroxylase; CYP27A1, sterol 27-hydroxylase; DAPK2, death-associated protein kinase 2; DHCR24, 24-dehydrocholesterol reductase; DRP1, dynamin-related protein 1; FAS, fatty acid synthase; FIS1, fission protein 1; FXR, farnesoid X receptor; GADD45G, growth arrest and DNA damage inducible gamma; HMGCS1, 3-hydroxy-3-methylglutaryl-CoA synthase 1; MFN1, mitofusin 1; MFN2, mitofusin 2; MTOR, mammalian target of rapamycin; MSMO1, methylsterol monooxygenase 1; ND1, NADH dehydrogenase subunit 1; NTCP, odium taurocholate cotransporting polypeptide; ULK1, unc-51 like autophagy activating kinase 1; OPA1, optic atrophy 1; PGC-1α, PPARγ coactivator-1α; PITPNC1, phosphatidylinositol transfer protein cytoplasmic 1; PIGR, polymeric immunoglobulin receptor; SREBF1, sterol regulatory element binding transcription factor 1; SREBF2, sterol regulatory element binding transcription factor 2; TRIM15, tripartite motif containing 15; VCAM1, vascular cell adhesion molecule 1

**Table S3** Effects of DON and DDE on the pH of ileal and colonic digesta in piglets

| **Items** | **CON** | **DON** | **DDE** | **SEM** | ***P*-value** |
| --- | --- | --- | --- | --- | --- |
| Ileum | 6.04 | 6.03 | 6.02 | 0.087 | 0.996 |
| Colon | 5.68 | 5.64 | 5.71 | 0.075 | 0.948 |

**Fig. S1** DON content in the liver

**Fig. S2** The concentration of GLCA in the liver was determined using LC‑MS/MS
