# Supplementary material for: Alginate-encapsulated enzymes mitigate deoxynivalenol-induced hepatic mitochondrial dysfunction and glycolithocholic acid accumulation in piglets
Source: J Anim Sci Biotechnol. 2026 Sep 5;17:170. doi: 10.1186/s40104-026-01484-x (PMC13545767; doi:10.1186/s40104-026-01484-x)
Supplement: Supplementary file 2 [file 40104_2026_1484_MOESM2_ESM.docx]

**Fig. S1. Gels and blots images**
